# Genome-wide characterization of the *bHLH* gene family in *Gynostemma pentaphyllum* reveals its potential role in the regulation of gypenoside biosynthesis

**DOI:** 10.1186/s12870-024-04879-y

**Published:** 2024-03-20

**Authors:** Yanhong Qin, Jinmei Li, Jianhua Chen, Shaochang Yao, Liangbo Li, Rongshao Huang, Yong Tan, Ruhong Ming, Ding Huang

**Affiliations:** 1 College of Pharmacy, Guangxi University of Chinese Medicine, Nanning, 530200 China; 2 Guangxi Key Laboratory of Zhuang and Yao Ethnic Medicine, Guangxi University of Chinese Medicine, Nanning, 530200 China

**Keywords:** *Gynostemma pentaphyllum*, Basic helix-loop-helix, Genome-wide characterization, Transcription factor, Gypenoside biosynthesis

## Abstract

**Background:**

*Gynostemma pentaphyllum*, an ancient Chinese herbal medicine, serves as a natural source of gypenosides with significant medicinal properties. Basic helix-loop-helix (bHLH) transcription factors play pivotal roles in numerous biological processes, especially in the regulation of secondary metabolism in plants. However, the characteristics and functions of the *bHLH* genes in *G. pentaphyllum* remain unexplored, and their regulatory role in gypenoside biosynthesis remains poorly elucidated.

**Results:**

This study identified a total of 111 bHLH members in *G. pentaphyllum* (GpbHLHs), categorizing them into 26 subgroups based on shared conserved motif compositions and gene structures. Collinearity analysis illustrated that segmental duplications predominately lead to the evolution of GpbHLHs, with most duplicated *GpbHLH* gene pairs undergoing purifying selection. Among the nine gypenoside-related *GpbHLH* genes, two GpbHLHs (*GpbHLH15* and *GpbHLH58*) were selected for further investigation based on co-expression analysis and functional prediction. The expression of these two selected GpbHLHs was dramatically induced by methyl jasmonate, and their nuclear localization was confirmed. Furthermore, yeast one-hybrid and dual-luciferase assays demonstrated that GpbHLH15 and GpbHLH58 could bind to the promoters of the gypenoside biosynthesis pathway genes, such as *GpFPS1*, *GpSS1,* and *GpOSC1*, and activate their promoter activity to varying degrees.

**Conclusions:**

In conclusion, our findings provide a detailed analysis of the bHLH family and valuable insights into the potential use of GpbHLHs to enhance the accumulation of gypenosides in *G. pentaphyllum*.

**Supplementary Information:**

The online version contains supplementary material available at 10.1186/s12870-024-04879-y.

## Introduction

The basic helix-loop-helix (bHLH) transcription factors (TFs) constitute one of the largest and most important families in eukaryotes, distinguished by their highly conserved bHLH domain [1]. The bHLH domain encompasses approximately 60 amino acids, which are constituted by two functionally distinct regions [2]. The N-terminal basic amino acid region, which varies from 10 to 15 amino acids in length and is essential for binding to the E-box (CANNTG) motif. Conversely, the C-terminal helix-loop-helix (HLH) structural domain, composed of two amphipathic alpha helices linked by a variable-length loop of approximately 40–50 amino acid residues, plays a pivotal role in facilitating homodimeric or heterodimeric complex formations and promoting protein–protein interactions [3, 4]. Additionally, plant MYC-like bHLH proteins contain an additional N-terminal MYC domain and a MYB interacting region, facilitating interactions with bHLH and R2R3-MYB domain proteins, respectively [5, 6].

Extensive research on bHLH TFs in plants has revealed their critical roles in numerous essential physiological processes. These include the transcriptional regulation of key developmental processes, such as stomata differentiation [7], seed coat differentiation [8], trichome/root hair formation [9], fruit dehiscence, and carpel margin development [10]. Additionally, some bHLH TFs are involved in seedling photomorphogenesis [11]. Furthermore, accumulating evidence suggests that bHLH proteins participate in the response to various abiotic stresses, enhancing plant stress tolerance, including tolerance to drought, salinity, low temperature, and mechanical damage [12–15]. Moreover, the regulatory roles of bHLH proteins in the secondary metabolites biosynthesis, such as phenolic acids [16], flavonoids [17, 18], alkaloids [19], and terpenoids [20, 21], have been well characterized in plants.

*Gynostemma pentaphyllum* (Thunb.) Makino, an herbaceous perennial plant of the Cucurbitaceae family, has a rich history in traditional Chinese medicine and is widely consumed as an herbal tea in Asian countries for its potential to prevent obesity [22]. The leaves of *G. pentaphyllum* plants, particularly the tender leaves, are the primary site for the synthesis of gypenosides, a class of dammarane-type triterpenoid saponins [23]. Gypenosides derived from *G. pentaphyllum* offer a wide range of health benefits, such as anti-cancer, anti-obesity, anti-inflammatory, and sedative-hypnotic effects [24–26]. The gypenoside content in *G. pentaphyllum* plants is a primary quality trait, making it imperative to understand the mechanism underlying gypenoside formation for developing high-yield gypenoside-producing plants. The gypenoside biosynthesis pathway has been extensively characterized, with four key enzyme-encoding genes, namely, *farnesyl diphosphate synthase* (*GpFPS1*), *squalene synthase* (*GpSS1*), *squalene epoxidase* (*GpSE2*), and *2,3-oxidosqualene cyclase* (*GpOSC1*), playing pivotal roles [23]. Moreover, the overexpression or interference with any of these genes (*FPS*, *SS*, *SE*, and *OSC*) has been demonstrated to significantly impact the production of dammarane-type triterpenoid saponins [27–30].

Given that the overexpression of bHLH TFs has been demonstrated to effectively regulate the selective biosynthesis of specific secondary metabolism in plants [31], investigating the regulatory roles of *bHLH* genes in gypenoside biosynthesis offers promising avenues for increasing gypenosides content in *G. pentaphyllum*. In this study, we identified 111 *GpbHLH* genes using a publicly available genomic database and carried out an exhaustive analysis of the bHLH family in *G. pentaphyllum*. Our analysis encompassed protein properties, phylogenetic relationships, gene structure, conserved domains, motifs composition, *cis*-acting elements, and *bHLH* expression patterns. The comprehensive analysis identified two bHLH proteins (GpbHLH15/58) as potential regulators of gypenoside biosynthesis. Their expression levels increased significantly under methyl jasmonate (MeJA) treatment, localizing primarily within the nucleus. Yeast one-hybrid (Y1H) and dual-luciferase assays demonstrated that GpbHLH15/58 could bind to the promoters of key enzyme-encoding genes (*GpFPS1*, *GpSS1*, and *GpOSC1*) in gypenosides biosynthetic pathway, and activate their expression to varying degrees. In summary, our findings expand on the understanding of the *bHLH* gene family in *G. pentaphyllum* and shed light on the underlying molecular mechanisms of gypenoside regulation, offering promising strategies for the improvement of gypenosides content.

## Results

### Identification and characterization of bHLH TFs in *G. pentaphyllum* and their evolutionary relationship

To comprehensively identify bHLH members in *G. pentaphyllum* at the genome-wide level, we employed *Arabidopsis* bHLH proteins as queries to search the *G. pentaphyllum* protein database. Additionally, we conducted a Hidden Markov Model search using the bHLH domain to globally search the *G. pentaphyllum* genome. Subsequently, the presence of the bHLH conserved domain was verified, and protein sequences lacking the bHLH domain were excluded. After removing the redundant sequences, we identified a total of 111 putative *GpbHLH* genes. For consistency, these *bHLH* genes were renamed *GpbHLH1*–*GpbHLH111* based on their chromosomal locations. Detailed information about these 111 *GpbHLH* genes, including gene name, gene ID, protein length, theoretical isoelectric point (pI), molecular weight (MW), and subcellular localization, is presented in Table S1. The proteins encoded by the 111 *GpbHLH* genes ranged in length from 91 to 1077 amino acids, with PI values ranging from 4.76 (*GpbHLH83*) to 10.57 (*GpbHLH74*) and MW values spanning from 10.18 (*GpbHLH18*) to 119.76 (*GpbHLH38*) kDa. Predictions using the Cell-PLoc indicated that the majority of GpbHLH proteins were localized in the nucleus, although a few were predicted to be extracellular, suggesting potential diverse tissue-specific expressions and functional roles among these *GpbHLH* genes.

**Fig. 1:**
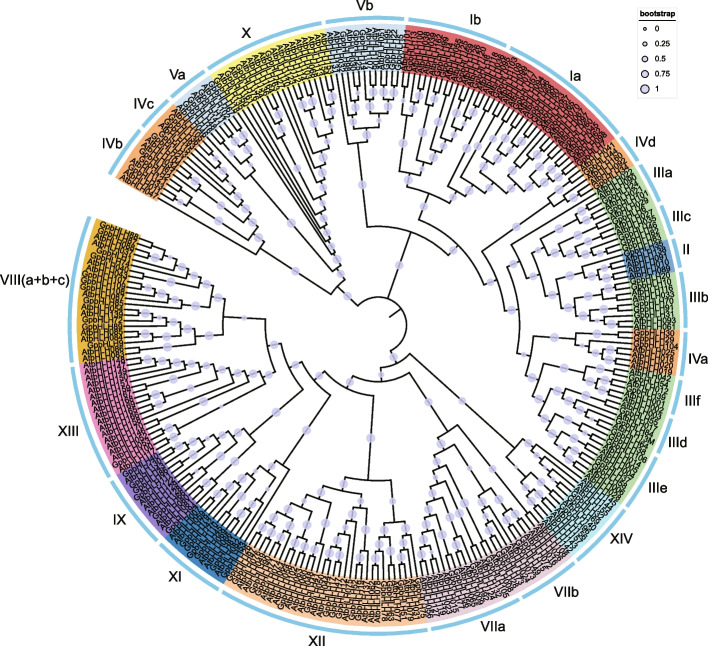
Phylogenetic relationship of bHLH proteins between *Gynostemma pentaphyllum* and *Arabidopsis thaliana*. The unrooted evolutionary tree was built by using the FastTree software with the maximum likelihood (ML) method. The reliability of each node was determined using the Shimodaira–Hasegawa test, which is highly correlated with commonly used bootstrap values. The 111 GpbHLH proteins are classified into 26 subfamilies, in line with the classification method of the 167 AtbHLH proteins, represented by different colored subgroups within the tree. Bootstrap values are visualized by a circle at each branch, with those below 50% not displayed in the figure

To elucidate the evolutionary relationships, we constructed an unrooted maximum likelihood (ML) phylogenetic tree based on bHLH protein sequences from *A. thaliana* and *G. pentaphyllum* (Fig. 1). The phylogenetic analysis clustered the *bHLH* genes into 26 well-conserved subgroups following the taxonomy proposed for *A. thaliana*. Among the 111 GpbHLH proteins, subgroup XII was the most abundant with 15 bHLH members, followed by subgroup Ia with nine members. Contrastingly, subgroups II and XIII contained the fewest GpbHLH proteins, each with only one member. Additionally, the number of genes in the specific subfamilies differed between *A. thaliana* and *G. pentaphyllum*. For example, subgroup XIII contained 17 *AtbHLHs* in *A. thaliana* but only one *GpbHLH* in the same subgroup, highlighting significant interspecific divergences with the *bHLH* gene family between the two species.

### Conserved domains, motif and gene structure analysis of the *GpbHLH* gene family

**Fig. 2:**
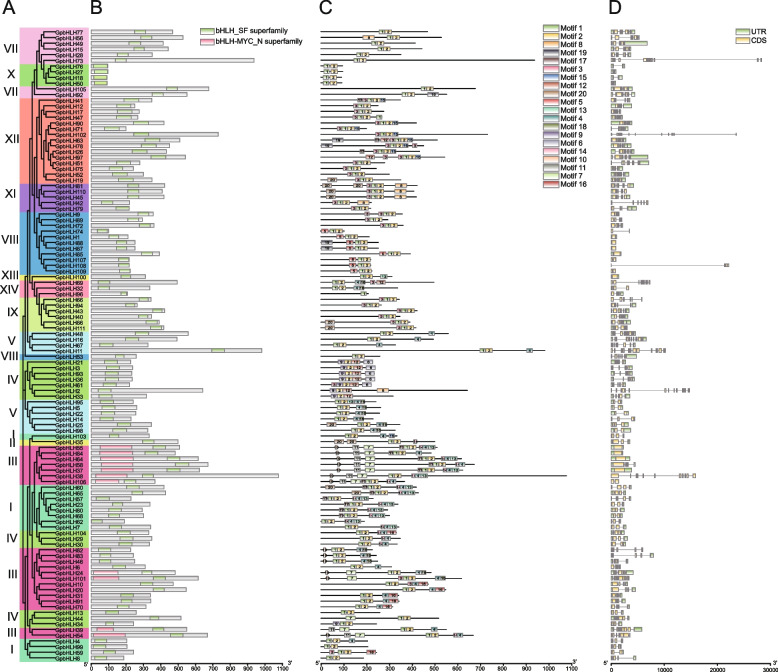
Analysis of conserved motifs, protein domains, and GpbHLH gene structures based on the phylogenetic relationships. **A** The phylogenetic tree of 111 GpbHLHs. **B** Conserved domains were identified in GpbHLH proteins, and their distributions were visualized using the TB tools software. **C** The conserved motifs distribution of GpbHLH proteins. The best matched sequences of each conserved motifs were provided in Supplementary Table S3. **D** Gene structure of *GpbHLH* genes. Green rectangles and yellow rectangles indicate the untranslated regions (UTRs) and exons, respectively. Introns are presented by black lines. The scale bar is indicated at the bottom of the diagram

To validate the findings from the phylogenetic analysis (Fig. 2A), we analyzed the conserved domains within the GpbHLH proteins. As shown in Fig. 2B, all GpbHLH proteins contained the bHLH domain. Notably, members of subgroups IIIa, IIId, IIIe, and IIIf possessed the MYC domain, in contrast to subgroups IIIb and IIIc. This observation is consistent with the notion that MYC-like bHLH TFs are distributed in the bHLH subfamily III in *A. thaliana* [32].

To gain insight into the sequence characteristics of GpbHLH proteins in *G. pentaphyllum*, we performed motif analysis using MEME, which identified a total of 20 conserved motifs (Table S2). GpbHLH proteins within the same phylogenetic group shared similar motif compositions and arrangements (Fig. 2C). For instance, subgroup IV was characterized by the presence of motifs 6, 9, and 12, while subgroup II contained two copies of motif 7, and subgroups IIId/e exhibited motifs 7, 10, 11, and 17. The presence of these specific motifs in particular subgroups underscores the relative conservation of motif compositions and arrangements within the same subgroup. Notably, motifs 1 and 2 were associated with the bHLH domain whereas motifs 7, 10, and 11 could form MYC structural domains individually (Fig. 2C).

To explore the exon/intron structure of the 111 *GpbHLH* genes, coding sequence (CDS)/intron patterns were visualized using feature structure of a generic file format (GFF) file (Fig. 2D). *GpbHLH* genes exhibited variable exon numbers, ranging from 1 to 15. Additionally, *GpbHLH* genes in the same subgroup presented highly similar gene structures but different among different subfamilies. For example, subgroup I contained *GpbHLH* genes with two or three exons, whereas subgroup X harbored genes with two exons. Consequently, a majority of the *GpbHLH* genes in the same subgroup exhibited similar motif compositions, gene structures, and conserved domain distribution, which further validated the evolutionary relationship analysis results.

### Chromosomal distribution, gene duplication, and synteny analysis of *GpbHLH* genes

**Fig. 3:**
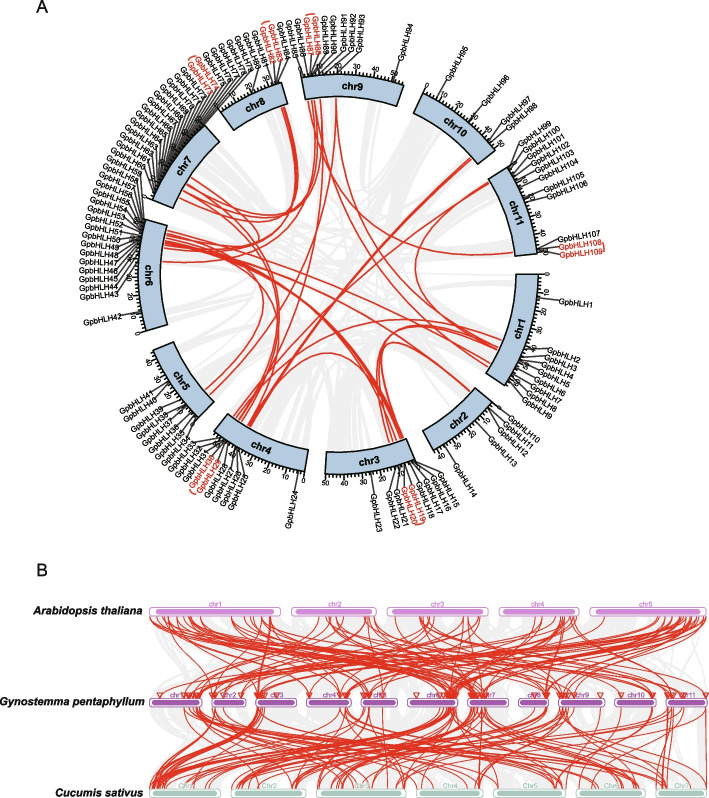
Distribution and collinearity analysis of *GpbHLH* genes. **A** Chromosomal locations and the intra-and inter-species collinear analysis of *bHLH* genes. The red lines inside the circle indicate segmentally duplicated *GpbHLH* gene pairs, while the tandem duplication gene pairs are labeled in red. **B** Inter-species collinear analysis of *bHLH* genes among *G. pentaphyllum*, *A. thaliana,* and *C. sativus*. Gray lines represent all collinear blocks, whereas syntenic *bHLH* gene pairs are highlighted as red lines

An analysis of the genomic locations revealed that the 111 *GpbHLH* genes were widely distributed across 11 chromosomes of *G. pentaphyllum* (Fig. 3A). Interestingly, the number of *GpbHLH* genes on each chromosome showed no correlation with chromosome length. Specifically, chromosome 7 hosted the largest number of genes, with 20 genes (*GpbHLH60*–*GpbHLH79*), followed by chromosome 6 (18 *GpbHLH* genes). In contrast, chromosome 10 had only two *GpbHLH* genes.

Gene duplication events are recognized as major drivers of genetic diversity in higher plants, contributing to species evolution. To explore whether gene duplication events contributed to the evolution of the *GpbHLH* gene family, a synteny analysis was performed (Fig. 3A). Notably, only six tandem duplication clusters were identified on the *G. pentaphyllum* chromosome, whereas 31 pairs of duplicated segments were identified within the *GpbHLH* gene family (Table S3), suggesting that segmental duplication events likely played a significant role in expanding the *GpbHLH* gene family. Additionally, the non-synonymous (Ka) and synonymous (Ks) substitution ratios for the duplicated gene pairs were calculated to assess the selective pressure during evolution (Table S3). All the segmentally and tandemly duplicated *GpbHLH* gene pairs exhibited Ka/Ks values < 1, indicating that these *GpbHLH* genes primarily underwent purifying selection.

Furthermore, we conducted interspecific collinearity analysis to identify orthologous *bHLH* genes among different species (Fig. 3B). It was observed that 99 *GpbHLH* genes exhibited collinearity relationships with 87 *bHLH* genes in *A. thaliana* and 96 *bHLH* genes in *Cucumis sativus*. The numbers of orthologous pairs between *G. pentaphyllum* and *A. thaliana*, and *G. pentaphyllum* and *C. sativus* were 128 and 151, respectively (Table S4). These results suggest that *GpbHLH* genes likely share functional similarities with *bHLH* genes in *C. sativus*.

### Gypenoside-related *GpbHLH* genes and their *cis*-element analysis

**Fig. 4:**
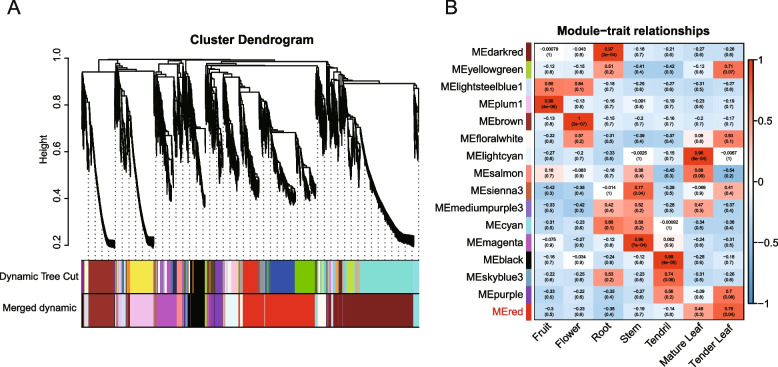
Co-expression network showing gypenoside-related module identified by WGCNA. **A** Hierarchical clustering tree (cluster dendrogram) showing 16 modules of co-expressed genes with color annotation. **B** Correlation between each module and genes expressed in different tissues. Pearson correlation coefficients and *p* values (in brackets) are indicated in the grid. A low to high degree of Pearson correlation coefficient between a specific module and the tissue is indicated by a change in color from blue to red

Gypenosides are synthesized in the leaves of *G. pentaphyllum*, especially in tender leaves [33, 34]. In this study, based on gene expression profiling, the expression patterns of gypenoside biosynthesis pathway genes (*GpFPS1*, *GpSS1*, *GpSE2,* and *GpOSC1*) were highly expressed in the tender leaf (Table S5), suggesting that tender leaf was the main tissue for biosynthesis of gypenosides skeletons in *G. pentaphyllum*. To identify bHLH TFs that play roles in regulating tender leaf-specific gypenoside biosynthesis, weighted gene co-expression network analysis (WGCNA) was conducted on seven different tissues (Fig. 4A). A total of 16 modules with similar expression patterns were identified (Fig. 4B). Furthermore, the MEred module shown positive correlation with tender leaf (*r* > 0.75), followed by mature leaf (*r* > 0.45), was regarded as a gypenoside-related module. In the gypenoside-related module, a total of 1774 genes including four gypenoside biosynthesis pathway genes (*GpFPS1*, *GpSS1*, *GpSE2,* and *GpOSC1*). In addition, nine *GpbHLH* genes were also identified in the gypenoside-related module. The FPKM value and WGCNA module of each gene is provided in Table S5. These findings suggest that the nine *GpbHLH* genes may be involved in the regulation of gypenoside biosynthesis.

**Fig. 5:**
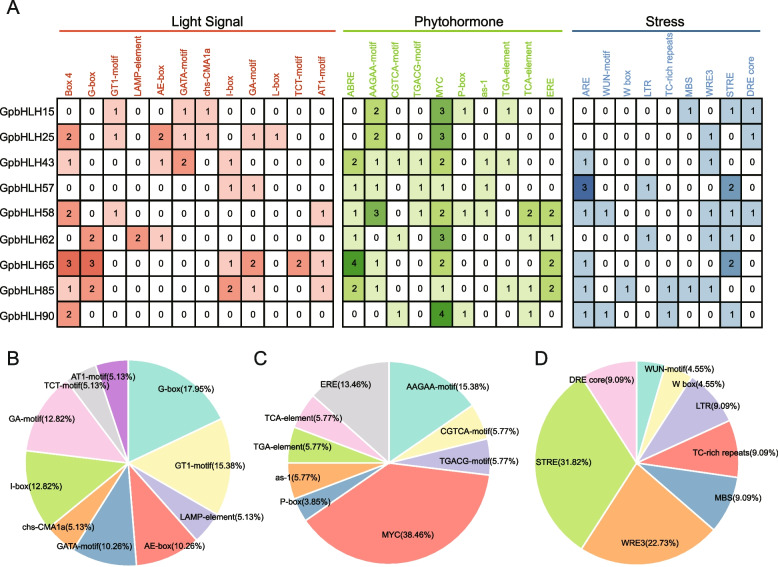
Promoter *cis*-elements analysis of the nine gypenoside-related *GpbHLH* genes. **A** The identified *cis*-acting elements were divided into three functional groups and represented by red, green and blue shades of colors. The numbers indicate the quantity of corresponding *cis*-acting elements in each *GpbHLH* gene promoter region. Distribution of *cis*-acting elements with different categories within each group of *cis*-acting elements with different specific functions, including light signal (**B**), hormone response (**C**), and stress response (**D**)

To analyze the potential *cis*-elements of the nine gypenoside-related *GpbHLH* genes, we extracted 2000 bp upstream promoter region sequences and employed the PlantCARE online tool for *cis*-acting element prediction (Fig. 5A). In total, 152 cis-acting elements were identified and categorized into three groups based on their functions: light signal responses (49 elements), phytohormone responses (70 elements), and stress responses (33 elements) (Table S6). In the light signal responses group, the most prevalent *cis*-element was box4, accounting for 25.92%, followed by G-box at 12.96% (Fig. 5B). The *cis*-elements were associated with responses to various plant hormones, such as abscisic acid (ABA), jasmonic acid, auxin, gibberellin, salicylic acid, and ethylene. Among these, CGTCA-motif, TGACG-motif, and MYC-motif were the most widely distributed MeJA-responsive elements (40.62%), while ABRE (ABA-responsive element) and AAGAA-motif represented ABA-responsive elements at 31.25% (Fig. 5C). Among the *cis*-elements involved in stress responses, some functioned in response to specific stress conditions. For instance, anaerobic induced response element (ARE) was essential for anaerobic induction, accounting for 26.67%. Additionally, wound responsiveness element (WRE3), low-temperature responsiveness (LTR), and dehydration responsive element (DRE) were also identified as stress responses (Fig. 5D). In summary, our findings suggest that the nine gypenoside-related *GpbHLH* genes play important roles in various biological processes, especially light signal response, hormone response, and stress response.

### Prediction of GpbHLHs involved in the regulation of gypenoside biosynthesis

**Fig. 6:**
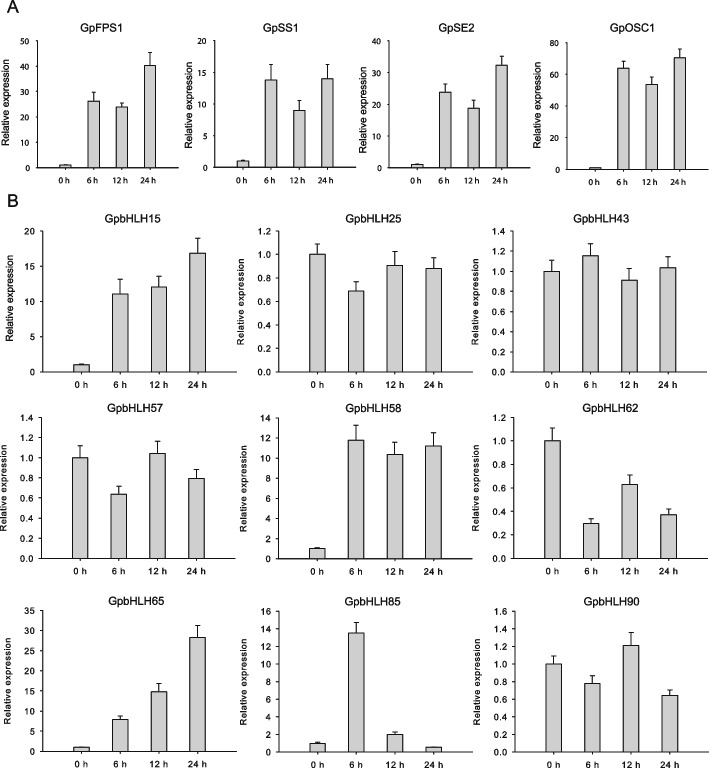
Expression patterns of gypenoside biosynthesis pathway genes and nine gypenoside-related *GpbHLH* genes under MeJA treatment. **A** The relative expression abundances of the four gypenoside biosynthesis pathway genes (*GpFPS1*, *GpSS1*, *GpSE2,* and *GpOSC1*) were determined using qRT-PCR in response to MeJA treatments. **B** The relative expression abundances of the nine gypenoside-related *GpbHLH* genes were determined using qRT-PCR in response to MeJA treatments. Values represent the mean ± SE (*n* = 3 biological replicates)

Gypenoside biosynthesis is speculated to be strongly induced by MeJA [35]. Moreover, the analysis of the nine gypenoside-related *GpbHLH* gene promoters revealed the presence of MeJA-responsive *cis*-elements, suggesting that these *GpbHLH* genes can be induced by MeJA. To verify this hypothesis, we measured the transcript levels of the four gypenoside biosynthesis pathway genes (*GpFPS1*, *GpSS1*, *GpSE2,* and *GpOSC1*) and the nine candidate *GpbHLH* genes in MeJA-treated leaves of *G. pentaphyllum*. As shown in Fig. 6A and B, *GpFPS1*, *GpSS1*, *GpSE2, GpOSC1*, *GpbHLH15*, *GpbHLH58*, *GpbHLH65*, and *GpbHLH85* exhibited strong induction by MeJA compared to the control. In addition, Pearson correlation analysis revealed strong associations between the four gypenoside biosynthesis pathway genes and two GpbHLHs (GpbHLH15/58) in response to MeJA (Fig. S1). Based on previous WGCNA results and the response of *GpbHLH* genes to MeJA, we speculate that *GpbHLH15*, *GpbHLH58* are likely involved in the regulation of MeJA-mediated gypenoside biosynthesis.

To evaluate the potential functions of these candidate GpbHLHs, we used the functional characteristics of homologous AtbHLHs in the same subgroup for prediction. In this study, *GpbHLH15* showed a high degree of homology to *Arabidopsis* PIF7 (AtbHLH072), which is known to serve as a regulatory hub that relays environmental signals to the plant transcriptional network [36]. Moreover, *GpbHLH58* is speculated to be a homolog of AtbHLHs involved in JA signal transduction, especially MYC2. *GpbHLH65* was correspondingly clustered with the AtbHLHs in subgroup Ia and predicted to be a homolog of AtbHLH067, which negatively regulates cold tolerance and interacts physically with ICE1 [37]. Lastly, *GpbHLH85* was grouped with AtbHLH037 (HEC2), AtbHLH043 (HEC3), and AtbHLH088 (HEC1), all of which are involved in stomata differentiation, transmitting tract formation, and stigma development [38] (Fig. 1). In addition to their roles in growth and development regulation, PIF and MYC2 homologs are known to regulate various secondary metabolites, including flavonoids and terpenoids [31, 39], suggesting that GpbHLH15 and GpbHLH58 are the most likely regulators of MeJA-mediated gypenoside biosynthesis. However, further evidence is required to confirm the hypothesis.

### Gypenoside biosynthesis genes were transcriptionally activated by GpbHLH15 and GpbHLH58

**Fig. 7:**
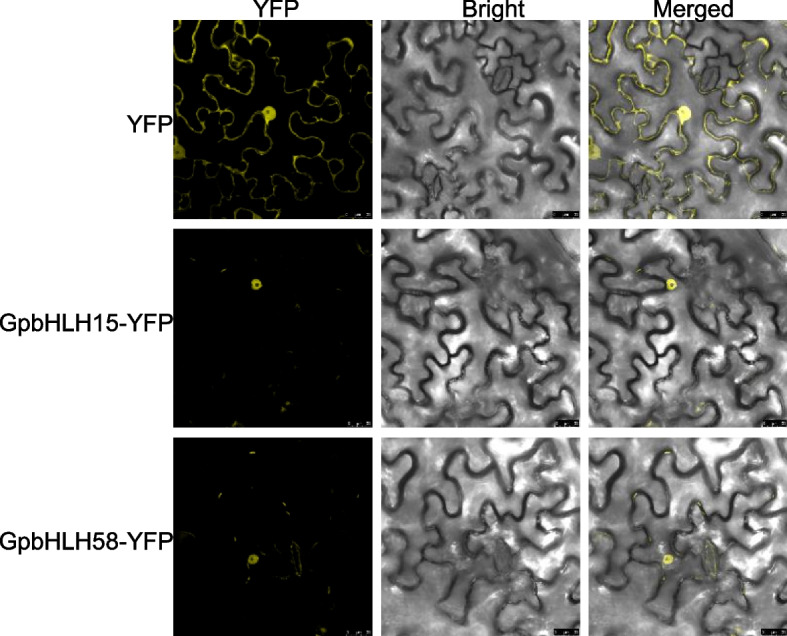
Subcellular localization of GpbHLH15 and GpbHLH58 proteins. The GpbHLH15/58-YFP fusion constructs were transfected into *N. benthamiana* leaves. YFP indicates the empty vector, which was used as a control. Confocal laser microscopy images were captured three days post-transfection. Scale bar = 25 μm

TFs typically function as transcriptional activators or repressors in the nucleus. To investigate the localization of GpbHLH15 and GpbHLH58 proteins, we conducted subcellular localization experiments in *N. benthamiana* leaves. As shown in Fig. 7, the yellow fluorescent protein (YFP) signals from the GpbHLH15-YFP and GpbHLH58-YFP fusion constructs were exclusively observed in the nucleus, indicating that GpbHLH15 and GpbHLH58 are both nuclear-localized TFs.

**Fig. 8:**
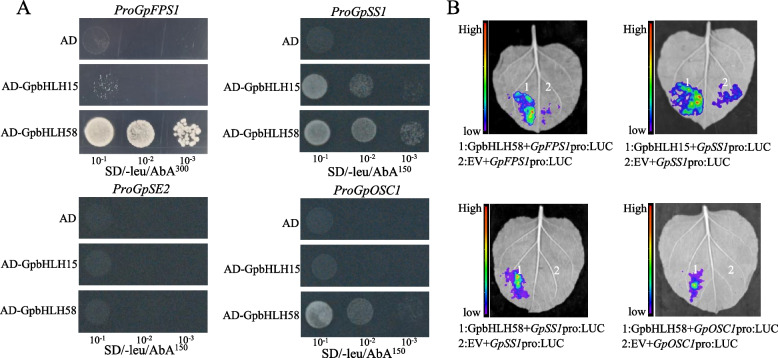
Interaction of GpbHLH15 and GpbHLH58 proteins with gypenoside biosynthesis gene promoters. **A** Y1H assays demonstrate the binding of GpbHLH15 to the *GpSS1* promoter, whereas GpbHLH58 exhibits binding to the promoters of *GpFPS1*, *GpSS1,* and *GpOSC1*. AD indicates a negative control using the pGADT7 empty vector. The transformed yeast cells were diluted thrice, with 10^–1^ dilutions each time and then spotted on the selective medium. **B** Dual-luciferase assays were performed using *GpFPS1*, *GpSS1,* and *GpOSC1* promoters driving the *Firefly luciferase* (*LUC*) gene as reporters, along with effectors (GpbHLH15, GpbHLH58). These results reveal that GpbHLH15 activates the promoter of *GpFPS1*, while GpbHLH58 promotes the expression of *GpFPS1*, *GpSS1*, and *GpOSC1*

In a previous study, bHLH-recognizing elements (BREs) (CANNTG) were identified in the promoters of four gypenoside biosynthesis enzyme genes (*GpFPS1*, *GpSS1*, *GpSE2*, and *GpOSC1*), implying that these genes could be potential targets of GpbHLHs [23]. To assess the ability of GpbHLH15 and GpbHLH58 to bind to the promoters of these four gypenoside biosynthesis enzyme genes, we utilized Y1H assays (Fig. 8A). Bit yeast cells co-transformed with the fusion vectors (AD-GpbHLH15 and AD-GpbHLH58) grew on the selective medium supplemented with *Aureobasidin A* (AbA). The results demonstrated that GpbHLH15 could bind to the promoter of *GpSS1 *in vitro, and the interactions between GpbHLH58 and the structural genes (*GpFPS1*, *GpSE2,* and *GpOSC1*) were also detected.

To further investigate the regulatory roles of GpbHLH15 and GpbHLH58 on *GpFPS1*, *GpSS1*, *GpSE2,* and *GpOSC1*, dual-luciferase assays were conducted (Fig. 8B). The results revealed that GpbHLH15 activated the expression of *GpSS1*, while GpbHLH58 and each of the firefly luciferase (LUC) reporter constructs driven by *GpFPS1*, *GpSS1,* and *GpOSC1* were co-infiltrated, higher levels of activation luciferase activities were observed. These findings demonstrate that GpbHLH15 activates the expression of *GpSS1*, whereas the GpbHLH58 protein activates the promoter activity of *GpFPS1*, *GpSS1,* and *GpOSC1*.

## Discussion

Recently, owing to its significant health benefits, the cultivation of *G. pentaphyllum* has gained significance in the agricultural industry. Moreover, the content of gypenosides is directly related to the plant’s quality and commercial value. However, there is considerable variation in the quality of *G. pentaphyllum*, which poses a challenge to the sustainable development of the industry. To address this issue, understanding the molecular mechanisms underlying gypenoside biosynthesis has become a current research highlight*.* The *bHLH* gene family, known for its vital role in various physiological processes [12, 40, 41], has been comprehensively studied in various plants. However, research on *bHLH* genes in *G. pentaphyllum* has been limited.

Here, we identified and characterized 111 *bHLH* genes in the *G. pentaphyllum* genome. While this number is similar to that reported in *C. mleo* (118), it is less than that in *C. sativus* (142), even though both plants belong to the Cucurbitaceae family [42, 43]. Notably, *G. pentaphyllum* has a larger genome size (582 Mb) than *C. sativus* (367 Mb) but possesses fewer *bHLH* genes, implying that the number of gene family members is not solely dependent on genome size but also on the species’ evolutionary history. The 111 GpbHLH proteins were classified into 26 subgroups, consistent with the classification system established for *A. thaliana* [2]. Furthermore, the highly conserved structural features of GpbHLH proteins within each subgroup corroborated the phylogenetic analysis. Thus, collectively, these findings strengthen the reliability of the GpbHLH classification and function prediction based on phylogenetic analysis.

Homology analysis is a valuable tool for predicting the functions of genes in various species. To date, numerous bHLH proteins in *A. thaliana* have been functionally characterized [32], thus enabling us to anticipate the roles of the bHLH gene family in *G. pentaphyllum*. For instance, bHLHs belonging to the subgroup IIIf are involved in anthocyanin biosynthesis, trichome and root hair formation [44–46], whereas members from the subgroup IIIb regulate stomata development and enhance freezing tolerance [37, 47]. Furthermore, bHLHs in subgroup XIII, including At159P1R2, At165PAR1, At166PAR2, At167P1R1, and At168P1R3, have been shown to control shade avoidance and auxin responses [48, 49]. Notably, bHLHs belonging to the subgroup XIII appear to have been significantly lost from the *G. pentaphyllum* genome compared to *A. thaliana*. These findings suggest that the shade avoidance-related gene family may have undergone adaptive evolution and the regulation of shade response may have been altered in *G. pentaphyllum*.

The production and modulation of secondary metabolites are important mechanisms that allow plants to adapt to environmental changes [50]. These processes involve complex signal transduction cascades in response to internal hormone signals and external environmental factors, ultimately leading to the activation or inhibition of downstream gene expression and the production of various specialized plant metabolites [51–53]. In our study, the *cis*-acting regulatory element prediction revealed the prevalence of *cis*-elements responsive to plant hormones, such as ABA and MeJA, among the nine gypenoside-related *GpbHLH* genes. Additionally, light-responsive *cis*-elements were widely identified. These findings suggest that gypenoside biosynthesis is influenced by various phytohormones, especially MeJA and ABA, as well as changing environmental conditions, especially light signals. However, further research is needed to comprehensively investigate the interplay among ABA, MeJA, and light signals in gypenoside biosynthesis regulation.

Based on previous studies, we hypothesized that *GpbHLH15* and *GpbHLH58* likely play a role in regulating gypenoside biosynthesis in *G. pentaphyllum*. The subcellular localization experiments revealed that GpbHLH15/58-YFP fusion proteins were localized in the nucleus (Fig. 5), indicating their regulatory function within the nucleus. Moreover, GpbHLH15 and GpbHLH58 could bind to the promoters of gypenoside biosynthesis genes and activate their promoter activity. In our experiments, the expression of GpbHLH58 (GpMYC2), along with gypenoside biosynthesis enzymes, was induced by extracellular MeJA treatment. A previous study reported that MeJA-induced AtMYC2 acted as a transcriptional activator in the MeJA signaling pathway [54]. Therefore, it is likely that GpbHLH58 functions as a transcriptional activator by binding to the promoters of gypenoside biosynthesis genes in a MeJA-dependent manner.

## Conclusions

This study conducted a comprehensive genome-wide characterization and analysis of the bHLH TF family in *G. pentaphyllum* and explored their potential roles in regulating gypenoside biosynthesis. Furthermore, we identified four gypenoside-related *GpbHLH* genes, with a particular focus on GpbHLH15 and GpbHLH58, as potential regulatory factors for gypenoside biosynthesis. Additionally, through Y1H and dual-luciferase assays, we demonstrated that GpbHLH15 and GpbHLH58 could bind to the promoters of enzyme-encoding genes in gypenoside biosynthetic pathway and activate their transcription, highlighting their involvement in the regulation of gypenoside biosynthesis. In conclusion, our findings provide valuable insights into the potential functions of GpbHLHs in the regulation of gypenosides in *G. pentaphyllum*.

## Materials and methods

### Plant materials and MeJA treatment

The *G. pentaphyllum* plant material used in this study is a variety whose genome was sequenced previously [23]. For MeJA treatment, six weeks old *G. pentaphyllum* plants were used, and then cultured in Hoagland’s nutrient solutions containing 100 μM MeJA. The MeJA-treated leaves were collected from three independent *G. pentaphyllum* plants before treatment (0 h) and 6, 12, 24 h after treatment.

### Identification of *GpbHLH* proteins in *G. pentaphyllum*

Firstly, to obtain putative GpbHLH members, bidirectional BLASTP comparisons were performed with an E-value of 10^–10^ by using the protein sequences of 167 AtbHLH as the query sequence. Secondly, the conserved structural domain of HLH (PF00010) was searched in the genome of *G. pentaphyllum* to identify the *GpbHLH* proteins by using HMMER (v3.3.2) with default parameters [55]. After excluding redundant sequences, a set of predicted bHLH candidate sequences were obtained. Finally, to confirm the presence of conserved bHLH domain in the candidate genes, Simple Modular Architecture Research Tool (SMART) (http://smart.embl-heidelberg.de/) was employed to filter with candidate genes.

### Sequence analysis, phylogenetic analysis and GpbHLH classification

According to the chromosomal position on the *G. pentaphyllum* chromosomes, all the identified GpbHLHs were renamed in order. The physical and chemical properties were determined by the ExPASy ProtParam (https://web.expasy.org/protparam/), comprising amino acid length, MW and predicted pI. Subcellular localization predictions were done with the online tool Cell-PLoc (v2.0) (http://www.csbio.sjtu.edu.cn/bioinf/Cell-PLoc/).

Multiple sequence alignments were performed using bHLH amino acid sequences of *G. pentaphyllum* and *Arabidopsis thaliana*, with ClustalX (v2.1) with the default parameters. After alignment the evolutionary tree was constructed by the FastTree software (v2.1.1), and the default parameters were adopted with the ML method [56]. The Interactive Tree of Life (iTOL) was used for visualisation and annotation of the phylogenetic tree [57]. For subfamily classification of GpbHLH proteins, the classification method of AtbHLH proteins was adopted, which was based on their evolutionary relationships [2].

### Conserved motif and gene structure analysis

For conserved motifs prediction of GpbHLH proteins, a motif discovery and analysis tool MEME (v5.1.1) [58] was used. The maximum motif number was set to 20. The Gene structure (exon/intron) of 111 *GpbHLH* genes were visualized by the TBtools software [59] based on feature structure information in GFF annotation files from the *G. pentaphyllum* genome. Additionally, for building a ML phylogenetic tree containing 111 GpbHLH protein sequences, FastTree software (v2.1.1) was used with default parameters.

### Genomic localization and gene duplication of *GpbHLH* genes

The chromosome distribution map of GpbHLH gene family members was drawn as described by Chen [59]. Comparison and annotation of orthologous *bHLH* genes among *G. pentaphyllum*, *A. thaliana* and *C. sativus* were completed using OrthoVenn2 [60]. Collinearity regions and gene duplication events of the bHLH gene family among different species were assessed as described previously [59]. Ka/Ks substitution of each duplicated *bHLH* genes was assessed using Tbtools software.

### Identification of co-expression modules

The clean reads of seven different tissue of *G. pentaphyllum* including root, stem, tender leaf, mature leaf, flower, tendril and fruit were downloaded from NCBI database (https://www.ncbi.nlm.nih.gov) under the accession number PRJNA720501 and PRJNA631355 in previous study [23, 61]. Then the clean reads were mapped to the reference genome using TBtools. The expression level was evaluated by normalization to fragments per kilobase of exon model per million mapped reads (FPKM) value calculated from the number of aligned reads for each gene. Weighted gene co-expression network analysis (WGCNA) package was used for co-expression analysis.

### Quantitative real-time PCR (qRT-PCR) validation

Total RNA extraction and reverse transcription were performed as described previously [35]. qRT-PCR was carried out using LightCycler 96 System (Roche). The *GpActin* gene was set as an internal control [61]. To calculate relative gene expression levels, the 2^−ΔΔCt^ analysis method was adopted. The primers used in this study are given in Supplementary Table S7.

### Subcellular localization

To perform subcellular localization analysis of GpbHLH proteins, the CDS of *GpbHLH15* and *GpbHLH58* were amplified and cloned into the pHB-YFP vector to form GpbHLH15/58-YFP fusion proteins. The GpbHLH15/58-YFP fusion constructs were transfected into *Nicotiana benthamiana* leaves via *Agrobacterium tumefaciens* strain GV3101 transformation. After 3 d transfection, the YFP signals were captured using a confocal laser scanning microscope (Zeiss LSM 510 Meta).

### Y1H assays

Y1H was performed as described previously [62]. To generate prey vectors, the CDS of *GpbHLH15* and *GpbHLH58* were cloned into pGADT7 plasmid. Meanwhile, the promoter fragments of four gypenoside biosynthetic gene, designated as *proGpFPS1*, *proGpSS1*, *proGpSE2* and *proGpOSC1*, containing the E-box *cis*-elements, were cloned into pAbAi plasmid to construct baits. The 10 × dilution series transformed yeast cells were dotted on the SD/-Leu plates with or without AbA.

### Dual luciferase assays

A transcriptional activity assay was conducted as described previously [62]. To generate effector vector, the CDS of *GpbHLH15* and *GpbHLH58* were cloned into pK2GW7 plasmid using the Gateway cloning system, while the promoter fragments of four gypenoside biosynthetic genes (*GpFPS1*, *GpSS1*, *GpSE2* and *GpOSC1*) were inserted into pGreenII 0800-LUC plasmid. Fluorescence was observed and captured using a fluorescence imaging system (IVIS Lumina LT, USA).

### Supplementary Information

**Additional file 1:** **Table S1. **Characteristics of the 111 bHLH genes in *Gynostemma pentaphyllum* of this study.

**Additional file 2:** **Table S2. **Analysis and distribution of conserved motifs in GpHLH proteins.

**Additional file 3:** **Table S3. **Segmentally and tandemly duplicated GpbHLH gene pairs.

**Additional file 4:** **Table S4. **One-to-one orthologous pairs of bHLH genes between two species.

**Additional file 5:** **Table S5.** The FPKM value of each structural gene from the pathway of gypenoside biosynthesis and bHLH genes identified in the gypenoside-related module.

**Additional file 6:** **Table S6. **Promoter analysis of the GpbHLH gene family in*Gynostemma pentaphyllum*.

**Additional file 7:** **Table S7. **List of oligonucleotide sequences used in this study.

**Additional file 8:** **Figure S1.** Correlations between the four gypenoside biosynthesis pathway genes and two GpbHLHs (GpbHLH15/58) in response to MeJA.

## Data Availability

The plant materials were collected from the orchard of Guangxi University of Chinese Medicine with the permission of the owner. The raw transcriptome data have been deposited in the NCBI database (https://www.ncbi.nlm.nih.gov) under the accession number PRJNA720501 and PRJNA631355 in previous study [23, 61].

## Abbreviations

bHLH: Basic helix-loop-helix
GpbHLHs: bHLH members in *G. pentaphyllum*
TF: Transcription factor
HLH: Helix-loop-helix
MeJA: Methyl jasmonate
Y1H: Yeast one-hybrid
pI: Isoelectric point
MW: Molecular weight
ML: Maximum likelihood
CDS: Coding sequence
GFF: Generic file format
Ka: Non-synonymous
Ks: Synonymous
WGCNA: Weighted gene co-expression network analysis
FPKM: Fragments per kilobase of exon model per million mapped reads
ABA: Abscisic acid
JA: Jasmonic acid
ABRE: ABA-responsive element
ARE: Anaerobic induced response element
WRE3: Wound responsiveness element,
LTR: Low-temperature responsiveness
DRE: Dehydration responsive element
YFP: Yellow fluorescent protein
BRE: bHLH-recognizing element
AbA: Aureobasidin A
qRT-PCR: Quantitative real-time PCR
UTR: Untranslated region
LUC: Firefly luciferase
