# Supplementary material for: Genome-wide characterization of the bHLH gene family in Gynostemma pentaphyllum reveals its potential role in the regulation of gypenoside biosynthesis
Source: BMC Plant Biol. 2024 Mar 20;24:205. doi: 10.1186/s12870-024-04879-y (PMC10953245; doi:10.1186/s12870-024-04879-y)
Supplement: Supplementary file 8 — Additional file 8: Figure S1. Correlations between the four gypenoside biosynthesis pathway genes and two GpbHLHs (GpbHLH15/58) in response to MeJA. [file 12870_2024_4879_MOESM8_ESM.docx]

**Genome-wide characterization of the *bHLH* gene family in *Gynostemma pentaphyllum* reveals its potential role in the regulation of gypenoside biosynthesis**

Yanhong Qin^1,2†^, Jinmei Li^1,2†^, Jianhua Chen^1,2^, Shaochang Yao^1,2^, Liangbo Li^1,2^, Rongshao Huang^1,2^, Yong Tan^1,2^, Ruhong Ming^1,2*^, Ding Huang^1,2*^

^1^College of Pharmacy, Guangxi University of Chinese Medicine, Nanning 530200, China

^2^Key Laboratory of Protection and Utilization of Traditional Chinese Medicine and Ethnic Medicine Resources of Guangxi Department of Education, Guangxi University of Chinese Medicine, Nanning 530200, China

^†^ **Yanhong Qin and Jinmei Li contributed equally to this work**

*** Corresponding author:**

Ruhong Ming, e-mail address: mingrh@gxtcmu.edu.cn, ORCID: 0000-0001-8449-1609

and Ding Huang, e-mail address: hdh016@126.com, ORCID: 0000-0003-0826-1101


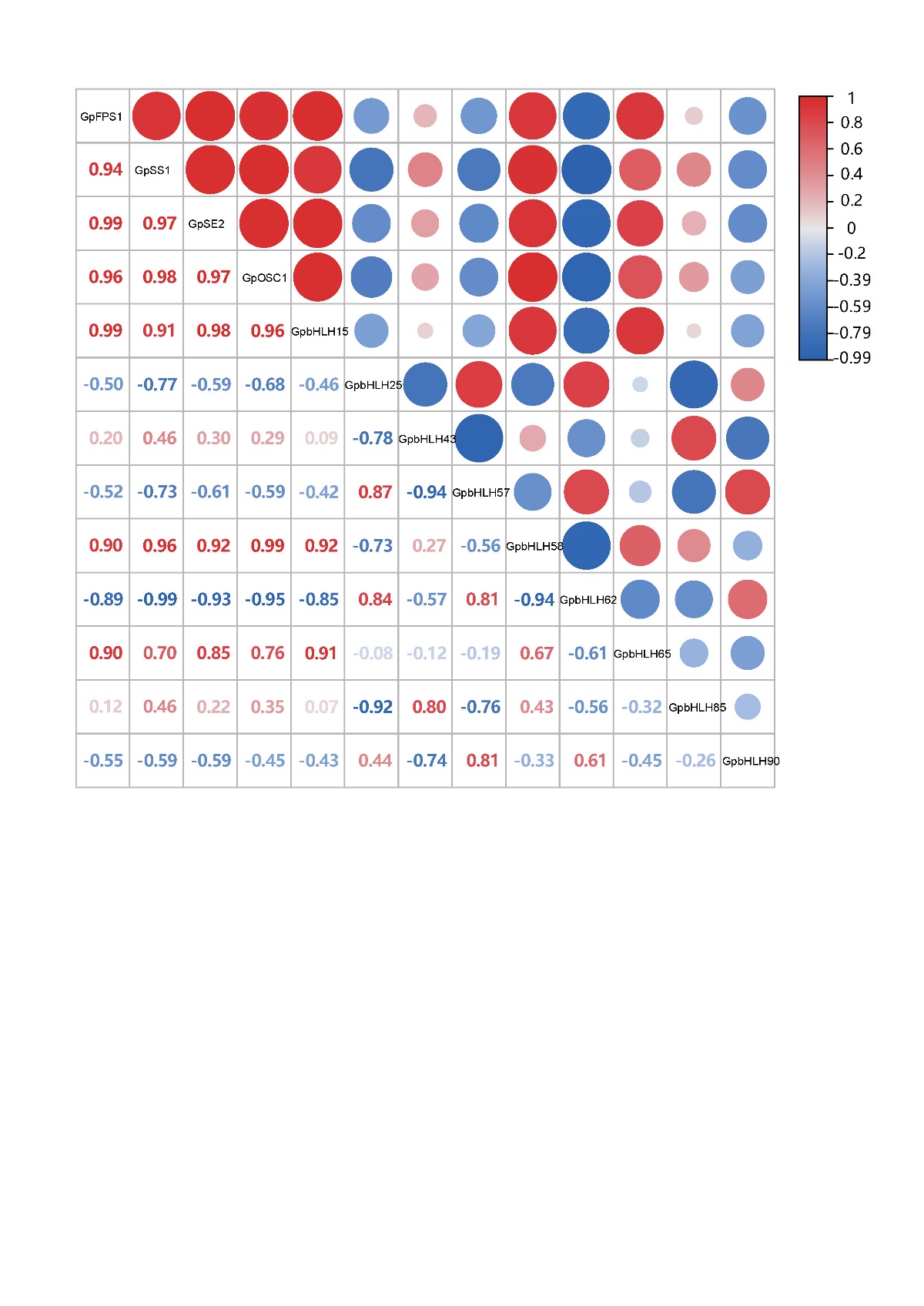


Figure. S1. Correlations between the four gypenoside biosynthesis pathway genes and two GpbHLHs (GpbHLH15/58) in response to MeJA.
